# Caesarean section for stillborn babies, Benin, Malawi, Uganda and United Republic of Tanzania

**DOI:** 10.2471/BLT.24.292424

**Published:** 2025-06-26

**Authors:** Maria del Rosario Alsina, Lenka Benova, Bianca Kandeya, Muzdalifat Abeid, Christian Agossou, Nicola Orsini, Effie Chipeta, Hussein Kidanto, Andrea Barnabas Pembe, Jean-Paul Dossou, Peter Waiswa, Aliki Christou, Claudia Hanson

**Affiliations:** a Department of Global Public Health, Karolinska Institutet, Tomtebodavägen 18, 17165 Solna, Sweden.; b Department of Public Health, Institute of Tropical Medicine, Antwerp, Belgium.; c Centre for Reproductive Health, Kamuzu University of Health Sciences, Blantyre, Malawi.; d Department of Obstetrics and Gynaecology, Aga Khan University, Dar es Salaam, United Republic of Tanzania.; e Centre de Recherche en Reproduction Humaine et en Démographie, Cotonou, Benin.; f Department of Obstetrics and Gynaecology, Muhimbili University of Health and Allied Sciences, Dar es Salaam, United Republic of Tanzania.; g Makerere University School of Public Health, Kampala, Uganda.

## Abstract

**Objective:**

To understand why caesarean sections are performed for stillborn babies by investigating caesarean section rates and indications in sub-Saharan African countries and to examine whether fetal vital status at admission is associated with caesarean section.

**Methods:**

The study involved registry data on 105 872 babies weighing 1000 g or more born to women aged 13 to 50 years at 16 hospitals in Benin, Malawi, Uganda and United Republic of Tanzania between 1 July 2021 and 30 June 2023. We assessed caesarean section rates and indications, and used multivariable logistic regression analyses to estimate associations between fetal heartbeat at admission and caesarean section, by birth outcome.

**Findings:**

The caesarean section rate was 28.0% (29 640/105 872) overall, 40.9% (858/2098) for intrapartum stillbirths and 19.0% (322/1694) for antepartum stillbirths. Previous caesarean section was among the top three indications across birth outcomes. Information on fetal heartbeat at admission was unavailable for 24.7% (7312/29 640) of caesarean section births. Multivariable analysis showed that the odds of a caesarean section was significantly higher when fetal heartbeat was not reported compared with the detection of a heartbeat among both antepartum (adjusted odds ratio, aOR: 2.55; 95% confidence interval, CI: 1.53–4.26) and intrapartum (aOR: 2.08; 95% CI: 1.51–2.87) stillbirths.

**Conclusion:**

Unknown fetal heartbeat at admission was associated with a higher odds of caesarean section, possibly due to attempts to provide optimum care given diagnostic uncertainty. Decision-making processes on the mode of birth need to be better understood and feasible fetal monitoring recommendations are required for low-resource settings.

## Résumé

**Objectif:**

Comprendre la raison des césariennes pour les bébés mort-nés en étudiant les taux et les indications de césarienne dans les pays d’Afrique subsaharienne et examiner si l’état vital du fœtus à l’admission est associé à la césarienne.

**Méthodes:**

Cette étude a porté sur les données de registres concernant 105 872 bébés pesant 1 000 g ou plus, nés de femmes âgées de 13 à 50 ans dans 16 hôpitaux du Bénin, du Malawi, de l’Ouganda et de la République-Unie de Tanzanie entre le 1^er^ juillet 2021 et le 30 juin 2023. Nous avons évalué les taux et les indications de césarienne et utilisé des analyses de régression logistique multivariables afin d’estimer les associations entre le rythme cardiaque fœtal à l’admission et la césarienne, par résultat de naissance.

**Résultats:**

Le taux de césarienne était de 28,0% (29 640/105 872) dans l’ensemble, de 40,9% (858/2 098) pour les mortinaissances intrapartum et de 19,0% (322/1 694) pour les mortinaissances antepartum. Les césariennes antérieures figuraient parmi les trois indications principales pour l’ensemble des résultats de naissance. Aucune information sur le rythme cardiaque fœtal à l’admission n’était disponible pour 24,7% (7 312/29 640) des naissances par césarienne. L’analyse multivariable a mis en évidence que la probabilité d’une césarienne était significativement plus élevée lorsque le rythme cardiaque fœtal n’était pas signalé, par comparaison avec la détection d’un rythme cardiaque parmi les mortinaissances aussi bien antepartum (odds ratio ajusté (aOR): 2,55; intervalle de confiance (IC) à 95%: 1,53–4,26) qu’intrapartum (aOR: 2,08; IC à 95%: 1,51–2,87).

**Conclusion:**

Un rythme cardiaque fœtal inconnu à l’admission était associé à un risque plus élevé de césarienne, peut-être en raison des tentatives d’apporter des soins optimaux compte tenu de l’incertitude diagnostique. Les processus décisionnels concernant le mode d’accouchement doivent être mieux compris, et des recommandations réalistes en matière de surveillance fœtale sont nécessaires pour les environnements à faibles ressources.

## Resumen

**Objetivo:**

Comprender por qué se realizan cesáreas en casos de mortinatos mediante la investigación de las tasas e indicaciones de cesárea en países del África subsahariana y examinar si el estado vital fetal al ingreso está asociado con la realización de cesáreas.

**Métodos:**

El estudio utilizó datos de registro de 105 872 nacimientos de bebés con un peso igual o superior a 1000 g, de mujeres de entre 13 y 50 años, en 16 hospitales de Benín, Malawi, Uganda y la República Unida de Tanzania entre el 1 de julio de 2021 y el 30 de junio de 2023. Se evaluaron las tasas e indicaciones de cesárea y se emplearon análisis de regresión logística multivariable para estimar la asociación entre la presencia de latido fetal al ingreso y la realización de cesáreas, según el resultado del parto.

**Resultados:**

La tasa global de cesárea fue del 28,0% (29 640/105 872); del 40,9% (858/2098) en casos de mortinatos intraparto y del 19,0% (322/1694) en mortinatos anteparto. La cesárea previa fue una de las tres principales indicaciones en todos los resultados del parto. La información sobre el latido fetal al ingreso no estaba disponible en el 24,7% (7312/29 640) de los nacimientos por cesárea. El análisis multivariable mostró que la probabilidad de cesárea fue significativamente mayor cuando no se informó el latido fetal, en comparación con su detección, tanto en mortinatos anteparto (razón de probabilidades ajustada [aOR]: 2,55; intervalo de confianza del 95%, IC: 1,53-4,26) como intraparto (aOR: 2,08; IC del 95%: 1,51-2,87).

**Conclusión:**

La ausencia de registro del latido fetal al ingreso se asoció con una mayor probabilidad de cesárea, posiblemente debido a intentos de proporcionar una atención óptima frente a la incertidumbre diagnóstica. Es necesario comprender mejor los procesos de toma de decisiones en relación con la vía del parto y formular recomendaciones viables para la monitorización fetal en contextos con recursos limitados.

## ملخص

الغرض فهم أسباب إجراء الولادة القيصرية للأطفال المولودين موتى من خلال تقصي معدلات الولادة القيصرية ومؤشراتها في دول جنوب الصحراء الكبرى الأفريقية، وفحص ما إذا كانت الحالة الحيوية للجنين عند دخوله المستشفى مرتبطة بالولادة القيصرية.

الطريقة شملت الدراسة بيانات التسجيل لـ 105872 طفلاً بوزن 1000 جرام أو أكثر، وُلدوا لنساء تتراوح أعمارهن بين 13 و50 عامًا في 16 مستشفى في كل من بنين، وملاوي، وأوغندا، وجمهورية تنزانيا المتحدة، في الفترة من 1 يوليو/تموز 2021 إلى 30 يونيو/حزيران 2023. وقمنا بتقييم معدلات الولادة القيصرية ومؤشراتها، واستخدمنا تحليلات التحوف اللوجستي متعدد المتغيرات، لتقدير الارتباط بين نبضات قلب الجنين عند دخوله المستشفى والولادة القيصرية، حسب نتائج الولادة.

النتائج بلغ معدل الولادة القيصرية %28.0 (29640/105872) إجمالاً، و%40.9 (858/2098) لحالات الوفاة أثناء الولادة، و%19.0 (322/1694) لحالات الوفاة قبل الولادة. وكان إجراء عملية قيصرية سابقة من بين أهم ثلاثة مؤشرات على نتائج الولادة. ولم تتوفر معلومات عن نبضات قلب الجنين عند دخول المستشفى في %24.7 (7312/29.640) من حالات الولادة القيصرية. أظهر التحليل متعدد المتغيرات أن احتمالات الولادة القيصرية كانت أعلى بشكل ملحوظ عند عدم تسجيل نبضات قلب الجنين، مقارنةً باكتشافها لدى كل من حالات الوفاة قبل الولادة (نسبة الاحتمالات المعدلة: 2.55؛ بفاصل ثقة مقداره %95: 1.53 إلى 4.26) وأثناء الولادة (نسبة الاحتمالات المعدلة: 2.08؛ ‏بفاصل ثقة مقداره %95: 1.51 إلى 2.87) مواليد متوفاة.

الاستنتاج ارتبط عدم معرفة نبضات قلب الجنين عند دخول المستشفى بارتفاع احتمالات الولادة القيصرية، ربما بسبب محاولات تقديم رعاية مثالية نظرًا لعدم اليقين التشخيصي. يجب فهم عمليات اتخاذ القرار بشأن طريقة الولادة بشكل أفضل، كما أن هناك حاجة إلى توصيات عملية لمراقبة الجنين في البيئات منخفضة الموارد.

## 摘要

**目的:**

旨在通过调查撒哈拉以南非洲国家的剖腹产率及其手术指征，来了解针对死胎实施剖腹产手术的原因，并检查入院时胎儿的生命状态是否符合剖腹产手术指征。

**方法:**

该项研究涉及了 2021 年 7 月 1 日至 2023 年 6 月 30 日期间在贝宁、马拉维、乌干达和坦桑尼亚联合共和国 16 家医院内出生的 105,872 名体重为 1,000 克或以上的婴儿（其母亲年龄介于 13 至 50 岁之间）的登记数据。我们评估了剖腹产率及其手术指征，并采用了多变量逻辑回归分析法以根据新生儿出生结局判断入院时的胎心状况与剖腹产手术之间的关联性。

**结果:**

总体剖腹产率为 28.0% (29,640/105,872)，其中针对产时死胎的剖腹产率为 40.9% (858/2,098)，而针对产前死胎的剖腹产率则为 19.0% (322/1,694)。既往剖腹产史是新生儿出生结局中排名靠前的三大手术指征之一。在通过剖腹产分娩的所有新生儿中，24.7% (7,312/29,640) 的新生儿的入院时胎心相关信息不可用。多变量分析结果显示，在产前【调整后比值比，aOR：2.55；95% 置信区间 (CI)：1.53-4.26】和产时（aOR：2.08；95% CI：1.51-2.87）死胎中，未报告胎心数据的孕妇接受剖腹产手术的几率明显高于那些接受过胎心监护的孕妇。

**结论:**

入院时胎心状况不明与剖腹产率提高相关联，这可能是由于医生考虑到诊断结果存在不确定性而试图为孕妇提供最佳护理所致。我们需要更深入地了解分娩方式相关决策制定过程，还需要为资源匮乏的地区提供切实可行的胎儿监测建议。

## Резюме

**Цель:**

Изучить причины выполнения кесарева сечения при мертворождении путем исследования частоты кесаревых сечений и показаний к ним в странах Африки южнее Сахары, а также определить, связан ли статус жизнеспособности плода при поступлении в больницу с последующим кесаревым сечением у матери.

**Методы:**

В исследовании использовались данные реестров по 105 872 младенцам массой 1000 граммов или более, рожденным женщинами в возрасте от 13 до 50 лет в 16 больницах Бенина, Малави, Объединенной республики Танзания и Уганды в период между 1 июля 2021 года и 30 июня 2023 года. Авторы оценили частоту кесарева сечения и показания к его выполнению. С помощью многофакторного логистического регрессионного анализа была оценена связь между наличием у плода сердцебиения при поступлении и последующим кесаревым сечением с распределением по исходу родов.

**Результаты:**

Общая частота кесарева сечения составила 28% (29 640/150 872), при этом 40,9% (858/2098) из них приходились на интранатальную гибель плода и 19,0% (322/1694) – на антенатальную. По всем итогам родов среди трех наиболее распространенных показаний было наличие кесарева сечения в анамнезе. Данные о наличии у плода сердцебиения при поступлении отсутствовали в 24,7% случаев родов кесаревым сечением (7312/29 640). Многофакторный анализ показал, что вероятность кесарева сечения была значительно выше в том случае, если у плода не наблюдалось признаков сердцебиения, по сравнению с выявлением сердцебиения как для случаев антенатального мертворождения (скорректированное отношение шансов, сОШ: 2,55; 95%-й доверительный интервал, ДИ: 1,53–4,26), так и для случаев интранатального мертворождения (сОШ: 2,08; 95%-й ДИ: 1,51–2,87)**.**

**Вывод:**

Отсутствие сведений о сердцебиении плода при поступлении ассоциировалось с более высокой вероятностью проведения матери кесарева сечения, возможно из-за попыток обеспечить оптимальный уход при неясном диагнозе. Процесс принятия решений о характере родоразрешения следует изучить глубже, и необходимы доступные способы мониторинга состояния плода в условиях недостаточных ресурсов.

## Introduction

Rising rates of caesarean section worldwide are a concern because of their potential short- and long-term effects.^1^ A population rate between 15% and 20% is generally viewed as sufficient to prevent negative perinatal outcomes for mothers and their babies.^2^ However, caesarean section rates currently exceed 20% globally and are expected to reach 30% by 2030, partly due to nonmedical indications, which account for 30% of indications in high- and middle-income countries where the reported rate ranges from 25% to 40%.^1^^,^^3^^,^^4^ In contrast, in sub-Saharan Africa the reported rate ranges from 3.5% to 6.6%, with previous caesarean section, malpresentation, malposition, cephalopelvic disproportion, fetal distress and other obstetric complications among the most frequently reported indications.^1^^,^^5^^–^^8^

Most reports on caesarean section rates and their main indications both globally and regionally have not included stillbirths in their estimates, which implies that annually over two million births worldwide were not considered.^1^^,^^9^^,^^10^ Consequently, little information exists about the rates and characteristics of caesarean sections for stillborn babies, which are relevant for regions where stillbirth rates are high, such as sub-Saharan Africa, which accounted for over 40% of all stillbirths worldwide in recent decades.^11^^,^^12^ Two facility-based studies conducted in Mozambique and Zanzibar, United Republic of Tanzania, in 2015 and 2016, respectively, found that caesarean section rates were higher among stillbirths than live births.^13^^,^^14^ Notably, both studies found that fetal heartbeat was not adequately monitored before a decision was made to perform a caesarean section.^13^^,^^14^

Birth by caesarean section is not recommended once fetal death has been confirmed, unless there is a maternal indication such as a hypertensive disorder or severe bleeding.^15^ Therefore, it is paramount that fetal health is assessed before deciding on the mode of birth to reduce the likelihood of an unnecessary caesarean section, particularly in sub-Saharan Africa, where maternal mortality and morbidity after caesarean section were among the highest globally in 2020.^4^ However, published studies found that there were operational challenges in implementing World Health Organization (WHO) fetal monitoring recommendations in low-resource settings, and that evidence was lacking on which monitoring techniques were the most effective in low- and middle-income countries.^16^^–^^18^

Given that knowledge about caesarean section for stillborn babies in sub-Saharan Africa is limited, our aims were to investigate caesarean section rates and indications, and to examine associations between fetal heartbeat ascertainment and caesarean section births for different birth outcomes in 16 hospitals across Benin, Malawi, Uganda and the United Republic of Tanzania.

## Methods

We used cross-sectional data from an electronic registry that was established for four hospitals in each of Benin, Malawi, Uganda and the United Republic of Tanzania as part of the action leveraging evidence to reduce perinatal mortality and morbidity (ALERT) research study.^19^^,^^20^ We included data collected between 1 July 2021 and 30 June 2023. Our study followed the Strengthening the Reporting of Observational Studies in Epidemiology statement for cross-sectional studies.^21^

The burden of maternal and perinatal mortality and morbidity is high in the study countries (Table 1), which were purposively selected to ensure a variety of health system characteristics. For each country, four hospitals with a high case load were included to represent a mix of public or private non-profit hospitals and district or referral hospitals. Details of the settings of, and the selection process for, these facilities were published in the ALERT study protocol.^19^ A summary of the characteristics of the 16 hospitals is provided in Table 1.

**Table 1:** Country and facility characteristics, study of caesarean section for stillborn babies, Benin, Malawi, Uganda and United Republic of Tanzania, 2021–2023

Characteristic	Country			
	Benin	Malawi	Uganda	United Republic of Tanzania
**Country**				
Income level^a^	Lower-middle	Low	Low	Lower-middle
Population caesarean section rate, %	5.3^b^	6.1^b^	5.3^b^	11^c^
Stillbirth rate, per 1000 total births^d^	20.0	16.1	15.1	18.3
Maternal mortality ratio, per 100 000 live births	523^e^	381^e^	284^e^	104^c^
Nursing and midwifery personnel, per 10 000 inhabitants (year)^f^	2.9 (2019)	7 (2020)	16.9 (2020)	5.5 (2018)
Medical doctors, per 10 000 inhabitants (year)^g^	0.6 (2019)	0.5 (2020)	1.6 (2020)	0.5 (2018)
**Facility**				
Total no. in study	4	4	4	4
Type				
Public	3	3	3	3
Private not-for-profit	1	1	1	1
No. of births in 2022^h^	12 106	19 441	15 443	8783
No. of facilities with a dedicated theatre for caesarean section	4	4	0	3
Main provider of caesarean sections	Doctors	Doctors and non-physician clinicians	Doctors	Doctors and non-physician clinicians
Method of fetal heartbeat assessment	Pinard stethoscope and fetal Doppler monitor	Pinard stethoscope	Pinard stethoscope and fetal Doppler monitor	Pinard stethoscope and fetal Doppler monitor
No. of Pinard stethoscopes per facility, range	3–8	0–4	2–3	1–4
No. of Doppler devices per facility, range	0–2	0	0–2	0–1

^a^ Data on country income levels were obtained from the World Bank.^22^

^b^ Data on population caesarean section rates for Benin, Malawi and Uganda were obtained from the Global Health Observatory.^23^

^c^ Data on the population caesarean section rate and maternal mortality ratio for the United Republic of Tanzania were obtained from the 2022 Demographic and Health Survey and Malaria Indicator Survey.^24^

^d^ Data on stillbirth rates were obtained from the United Nations’ Inter-agency Group for Child Mortality Estimation.^25^

^e^ Data on maternal mortality ratios for Benin, Malawi and Uganda in 2020 were obtained from the World Bank.^26^

^f^ Data on nursing and midwifery personnel were obtained from the World Health Organization.^27^

^g^ Data on medical doctors were obtained from the World Health Organization.^28^

^h^ Data for 1 January to 31 December 2022 were obtained from the action leveraging evidence to reduce perinatal mortality and morbidity (ALERT) electronic registry.

Our study included data on babies with a birth weight of 1000 g or more that were born in the included hospitals to women aged between 13 and 50 years. We excluded babies born outside participating facilities and admitted for postnatal care. In addition, we performed a complete case analysis and excluded participants for whom data were missing on birth outcomes, maternal or fetal characteristics, obstetric history, antenatal care or referral status.

### Data collection

The ALERT data registry includes information on perinatal health and care indicators extracted from maternal antenatal cards, hospital admission books, labour records and delivery and postnatal registers. In establishing the registry, maternity staff and data clerks were informed about the ALERT operational manual and trained to collect data using the Research Electronic Data Capture (REDCap) application (Vanderbilt University, Nashville, United States of America). Data were entered prospectively on site before women were discharged, which allowed data collectors to consult health workers if the information obtained from different data sources was contradictory. In addition, for our study the ALERT data management team incorporated completeness and consistency checks of the key variables used.

A three-level data management system was established to assure data quality: (i) hospital data controllers conducted daily completeness checks; (ii) country data managers oversaw data consistency and completeness each week; and (iii) an international coordination team held weekly meetings to monitor and address emerging data collection issues.^19^^,^^20^ Additionally, registry data were cross-validated against district-level data sets from the same facilities; concordance was strong.

We represented the mode of birth by a binary variable that indicated either: (i) birth by elective or emergency caesarean section; or (ii) an uncomplicated or assisted vaginal birth. The main independent variable was fetal heartbeat at the time of hospital admission. We coded the results of fetal heartbeat monitoring as positive, no heartbeat or no information (i.e. reported as not documented or missing).

We stratified the analysis by birth outcome, which was classified as either a live birth, an antepartum stillbirth or an intrapartum stillbirth. We adopted the International Classification of Diseases definition of stillbirth as, “the death, before or during labour, of a baby weighing 1000 grams or more and/or with a gestational age of 28 weeks or more.”^11^^,^^29^ Fetal appearance was used to establish the time of death. The observation of skin maceration by maternity staff, which indicates that death occurred 12 hours or more before the onset of labour, was used as a proxy for antepartum stillbirth; whereas a baby born dead without signs of skin maceration was classified as an intrapartum stillbirth.

Additional independent variables were selected on the basis of literature reports and clinical experience; they included variables reported in the registry that had an established relationship to birth outcome.^30^^,^^31^ These variables covered maternal, obstetric and fetal characteristics and were treated as potential confounders. A detailed list of confounders and their definitions is available in the online repository.^32^

### Data analysis

For the main analysis, data from the 16 hospitals were pooled. Descriptive statistics are used to report the frequencies and percentages of maternal, obstetric and fetal characteristics by mode of birth and the rate of, and indications for, caesarean section by birth outcome. Three multivariable logistic regression models were specified to study the association between fetal heartbeat at admission and caesarean section, stratified by birth outcome. First, we conducted *χ^2^* tests to explore associations between the birth outcome and each possible independent variable. Only variables that had a significant association (i.e. a *P*-value less than 0.05) with both the outcome and the main independent variable were considered for inclusion in the models. In addition, we performed a bivariate logistic regression analysis, and we included variables that had a *P*-value less than 0.25 or that were clinically relevant in the final multivariable models.^33^ We checked multicollinearity using a variance–covariance matrix of estimates with a correlation coefficient of 0.8 as the cut-off point.^34^ The fit of the model was assessed using the Pearson goodness-of-fit test and the area under the receiver operating characteristic curve, for which we considered a value of 0.7 or above as acceptable.^35^ Results are reported as adjusted odds ratios (aORs) with 95% confidence intervals (CIs). To complement the analysis, we also report the absolute risk of a caesarean section, which was defined as the number of cases divided by the total number of individuals within each category of the variables included in the model. All analyses were conducted using Stata v. 17 (StataCorp LLC, College Station, USA).

To assess the potential impact of stillbirth misclassification, we conducted a sensitivity analysis in which the antepartum stillbirth of a baby with a positive fetal heartbeat at admission was reclassified as an intrapartum stillbirth. In addition, a stratified analysis of caesarean section indications by parity was performed to explore differences in the pattern of indications between nulliparous and multiparous women. The results of these two analyses are presented in the online repository.^32^

The ALERT study received ethical approval from both local and national ethics committees in all participating countries.^19^

## Results

**Fig. 1:**
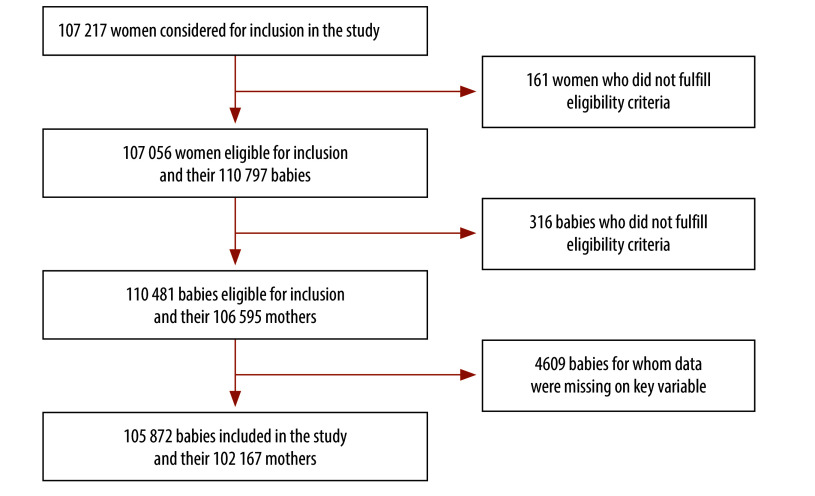
Participant inclusion, study of caesarean section for stillborn babies, Benin, Malawi, Uganda and United Republic of Tanzania, 2021–2023

Notes: Women were recruited between 1 July 2021 and 30 June 2023 at four hospitals each in Benin, Malawi, Uganda and the United Republic of Tanzania. Babies’ data on key variables were missing for: birth outcome (49 babies); maternal age (106 babies); number of previous pregnancies (698 babies); number of antenatal care visits (831 babies); previous caesarean section (66 babies); pregnancy complications (271 babies); referral status (176 babies); baby’s presentation (88 babies); birthweight (333 babies); and gestational age (1991 babies).

The analysis included data on 105 872 babies born to 102 167 women (Fig. 1); 3.5% of babies shared a mother. Of the 105 872, 29 640 (28.0%) were born by caesarean section and 76 232 (72.0%) were born vaginally.

Babies born by caesarean section tended to have mothers who were older than the mothers of those born vaginally (Table 2). In addition, their mothers attended more antenatal care visits but parity was similar in the two groups. Hypertensive disorders in the index pregnancy were more common among the mothers of babies born by caesarean section: 14.2% (4218/29 640) versus 4.9% (3720/76 232) among the mothers of babies born vaginally.

**Table 2:** Maternal, obstetric and fetal characteristics, by mode of birth, study of caesarean section for stillborn babies, Benin, Malawi, Uganda and United Republic of Tanzania, 2021–2023

Characteristic	Mode of birth, no. of births (%)	
	Caesarean section (*n* = 29 640)	Vaginal (*n* = 76 232)
**Maternal**		
Age, years		
< 20	4 696 (15.8)	17 946 (23.5)
20–29	16 104 (54.3)	39 001 (51.2)
30–39	8 048 (27.2)	17 317 (22.7)
≥ 40	792 (2.7)	1 968 (2.6)
Parity at index pregnancy		
Nulliparous	10 555 (35.6)	30 390 (39.9)
Multiparous (1–4 deliveries)	17 434 (58.8)	41 325 (54.2)
Grand multiparous (≥ 5 deliveries)	1 651 (5.6)	4 517 (5.9)
Outcome of previous pregnancy		
No previous pregnancy	9 590 (32.4)	28 450 (37.3)
Live birth	16 593 (56.0)	41 216 (54.1)
Miscarriage	2 403 (8.1)	5 023 (6.6)
Stillbirth	495 (1.7)	555 (0.7)
Neonatal death	400 (1.3)	543 (0.7)
Missing data	159 (0.5)	445 (0.6)
Number of antenatal care visits for index pregnancy		
0	276 (0.9)	1 060 (1.4)
1–3	8 972 (30.3)	28 459 (37.3)
4–7	19 194 (64.8)	44 789 (58.8)
≥ 8	1 198 (4.0)	1 924 (2.5)
Antenatal complications^a^		
None	24 642 (83.2)	69 549 (91.3)
Hypertensive disorder	4 218 (14.2)	3 720 (4.9)
Malaria	395 (1.3)	717 (0.9)
Severe anaemia	407 (1.4)	423 (0.6)
HIV infection	769 (2.6)	2 386 (3.1)
Other chronic condition^b^	325 (1.1)	346 (0.5)
**Obstetric**		
Multiple pregnancy	2 667 (9.0)	4 750 (6.2)
Fetal heartbeat at admission		
Positive heartbeat	21 402 (72.2)	71 928 (94.4)
No heartbeat	926 (3.1)	2 351 (3.1)
No information available	7 312 (24.7)	1 953 (2.6)
Fetal presentation		
Cephalic	26 669 (90.0)	73 569 (96.5)
Breech	2 630 (8.9)	2 578 (3.4)
Transverse	341 (1.2)	85 (0.1)
Induction or augmentation of labour	806 (2.7)	2 189 (2.9)
Referred from another facility	8 492 (28.7)	11 943 (15.7)
Maternal outcome at discharge		
No complications	28 495 (96.1)	75 369 (98.9)
Complications^c^	951 (3.2)	285 (0.4)
Death	120 (0.4)	135 (0.2)
Missing data	74 (0.2)	443 (0.6)
**Fetal**		
Birthweight		
Very low (1000–1499 g)	439 (1.5)	1 112 (1.5)
Low (1500–2499 g)	4 794 (16.2)	10 810 (14.2)
Normal (2500–3999 g)	23 411 (79.0)	62 452 (81.9)
Macrosomia (≥ 4000 g)	996 (3.4)	1 858 (2.4)
Gestational age		
Very preterm (< 32 weeks)	431 (1.5)	1 349 (1.8)
Moderate to late preterm (32–36 weeks)	4 235 (14.3)	9 484 (12.4)
Term (37–41 weeks)	23 724 (80.0)	62 862 (82.5)
Post-term (≥ 42 weeks)	1 250 (4.2)	2 537 (3.3)
5-minute Apgar score^d^		
Low (0–3 points)	1 467 (5.0)	3 083 (4.0)
Moderately abnormal (4–6 points)	888 (3.0)	1 493 (2.0)
Reassuring (7–10 points)	27 265 (92.0)	71 622 (94.0)
Sex		
Female	13 910 (46.9)	37 949 (49.8)
Male	15 725 (53.1)	38 272 (50.2)
Birth outcome		
Live birth	28 460 (96.0)	73 620 (96.6)
Intrapartum stillbirth	858 (2.9)	1 240 (1.6)
Antepartum stillbirth	322 (1.1)	1 372 (1.8)

HIV: human immunodeficiency virus.

^a^ Each pregnancy could have more than one complication.

^b^ Other chronic conditions included cardiac or renal disease, gestational diabetes and diabetes.

^c^ Maternal complications at discharge included postpartum infection, haemorrhage, hypertensive disorders and anaesthesia-related complications.

^d^ As Apgar scores were reported for both live and stillborn births, the denominator was the total number of births, consistent with other variables in the table.

No information on fetal heartbeat at admission was available for 24.7% (7312/29 640) of babies born by caesarean section, compared with 2.6% (1953/76 232) of those born vaginally. The rate of referrals from another facility was higher for babies born via caesarean section compared to those born vaginally, at 28.7% (8492/29 640) versus 15.7% (11 943/76 232), respectively; as was the rate of postnatal complications, at 3.2% (951/29 640) versus 0.4% (285/76 232), respectively. Only minor differences between these two groups were observed for all fetal characteristics, except birth outcome. There were 3792 stillbirths among the 105 872 births (3.6%), of which 55% (2098/3792) were intrapartum stillbirths. Although intrapartum stillbirths were more frequent among babies born by caesarean section (2.9%; 858/29 640) compared to vaginal births (1.6%; 1240/76 232), antepartum stillbirths were more common among babies born vaginally: 1.1% (322/29 640) among caesarean section deliveries versus 1.8% (1372/76 232) among vaginal births.

### Caesarean section rates and indications

The caesarean section rate was 40.9% (858/2098) for intrapartum stillbirths and 19.0% (322/1694) for antepartum stillbirths, compared with 27.9% (28 460/102 080) for live births. At least one indication was reported for 91.0% (26 960/29 640) of all caesarean section births (Table 3). Previous caesarean section was the most common indication among intrapartum stillbirths and live births: 26.1% (182/697) and 51.6% (9871/19 126), respectively. Among antepartum stillbirths, fetal death was the most frequently reported indication (34.5%; 111/322) and it was the only reported indication for 35.1% (39/111) of antepartum stillbirths delivered by caesarean section with fetal death as an indication. Antepartum haemorrhage was an indication for caesarean section among 14.3% (46/322) and 19.1% (164/858) of antepartum and intrapartum stillbirths, respectively, and prolonged labour was an indication among 14.6% (47/322) and 17.0% (146/858), respectively. Among nulliparous women, prolonged labour was the most frequent indication for those who had an intrapartum stillbirth (27.4%; 49/179) or a live birth (36.3%; 3739/10 295).

**Table 3:** Most-reported indications for caesarean section, by birth outcome, study of caesarean section for stillborn babies, Benin, Malawi, Uganda and United Republic of Tanzania, 2021–2023

Variable	Proportion of births by caesarean section, % (no./*n*)^a^		
	Birth outcome		
	Antepartum stillbirth	Intrapartum stillbirth	Live birth
**Caesarean section rate**	19.0 (322/1694)	40.9 (858/2098)	27.9 (28 460/102 080)
**Caesarean sections with a reported indication**	88.2 (284/322)	84.0 (721/858)	91.2 (25 955/28 460)
**Ten most-reported indications^b^**			
1	Fetal death (34.5%; 111/322)	Previous caesarean section (26.1%; 182/697)^c^	Previous caesarean section (51.6%; 9 871/19 126)^c^
2	Previous caesarean section (29.4%; 74/252)^c^	Antepartum haemorrhage (19.1%; 164/858)	Prolonged labour (26.0%; 7 400/28 460)
3	Prolonged labour (14.6%; 47/322)	Prolonged labour (17.0%; 146/858)	Fetal distress (14.4%; 4 090/28 460)
4	Antepartum haemorrhage (14.3%; 46/322)	Fetal distress (11.2%; 96/858)	Hypertensive disorder (9.0%; 2 548/28 460)
5	Hypertensive disorder (13.0%; 42/322)	Hypertensive disorder (10.5%; 90/858)	Malpresentation (8.0%; 2 271/28 460)
6	Malpresentation (12.4%; 40/322)	Fetal death (10.3%; 88/858)	Elective caesarean section (5.5%; 1 569/28 460)
7	Multiple pregnancy (7.8%; 25/322)	Malpresentation (8.7%; 75/858)	Multiple pregnancy (5.4%; 1 538/28 460)
8	Fetal distress (3.7%; 12/322)	Premature rupture of membranes (5.1%; 44/858)	Antepartum haemorrhage (2.5%; 718/28 460)
9	Elective caesarean section (3.7%; 12/322)	Cord complication (4.4%; 38/858)	Premature rupture of membranes (2.4%; 669/28 460)
10	Premature rupture of membranes (1.2%; 4/322)	Multiple pregnancy (3.1%; 27/858)	Post-term delivery (2.0%; 579/28 460)

^a^ Figures are for the percentage of births by caesarean section (number/total number of births by caesarean section for each birth outcome), except where otherwise indicated.

^b^ Each caesarean section could have more than one indication.

^c^ Figures are for women who had previously given birth.

### Fetal heartbeat and caesarean section

**Fig. 2:**
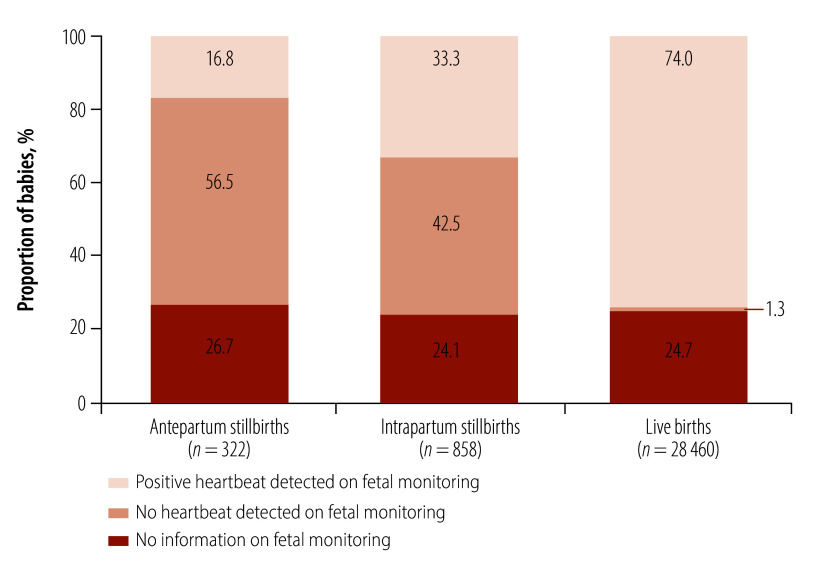
Fetal heartbeat at admission for babies born by caesarean section, by birth outcome, study of caesarean section for stillborn babies, Benin, Malawi, Uganda and United Republic of Tanzania, 2021–2023

No information was available on fetal heartbeat at admission for 24.7% (7312/29 640) of all caesarean section births; the proportion was similar for stillbirths and live births (Fig. 2). In the multivariable analysis, the adjusted odds of a caesarean section was significantly higher when fetal heartbeat was not reported compared with a positive heartbeat among both antepartum (aOR: 2.55; 95% CI: 1.53–4.26) and intrapartum (aOR: 2.08; 95% CI: 1.51–2.87) stillbirths (Table 4). Unadjusted odds ratios are reported in the online repository.^32^ The absolute risk of a caesarean section among antepartum stillbirths for which no information on fetal heartbeat was available at admission was 45.5% (86/189), and the corresponding absolute risk among similar intrapartum stillbirths was 61.6% (207/336). Information on adjusted absolute risks, which were estimated from model-predicted probabilities, is available in the online repository.^32^ In the sensitivity analysis, after 238 antepartum stillbirths of babies with a positive heartbeat at admission were reclassified as intrapartum stillbirths, the adjusted odds of a caesarean section when fetal heartbeat was not reported were even higher among both antepartum (aOR: 6.13; 95% CI: 4.01–9.36) and intrapartum (aOR: 2.44; 95% CI: 1.79–3.33) stillbirths. Among live births, the odds of a caesarean section was over nine times higher (aOR: 9.19; 95% CI: 8.63–9.78) when no information on fetal heartbeat was available at admission compared with a reported positive heartbeat; the corresponding absolute risk was 80.3% (7019/8740).

**Table 4:** Effect of fetal, maternal and demographic characteristics on the risk of caesarean section stratified by birth outcome, by multivariable logistic regression analysis, study of caesarean section for stillborn babies, Benin, Malawi, Uganda and United Republic of Tanzania, 2021–2023

Characteristic	Birth outcome							
	Antepartum stillbirth			Intrapartum stillbirth			Live birth	
	Caesarean section births as a proportion of all births, no./*n*(%)	Risk of caesarean section, aOR (95%CI)		Caesarean section births as a proportion of all births, no./*n*(%)	Risk of caesarean section, aOR (95%CI)		Caesarean section births as a proportion of all births, no./*n*(%)	Risk of caesarean section, aOR (95%CI)
**Fetal heartbeat at admission**								
Positive heartbeat	54/238 (22.7)	1.00 (reference)		286/700 (40.9)	1.00 (reference)		21 062/92 392 (22.8)	1.00 (reference)
No heartbeat	182/1267 (14.4)	0.47 (0.31–0.71)		365/1062 (34.4)	0.55 (0.44–0.70)		379/948 (40.0)	1.51 (1.29–1.75)
No information	86/189 (45.5)	2.55 (1.53–4.26)		207/336 (61.6)	2.08 (1.51–2.87)		7 019/8 740 (80.3)	9.19 (8.63–9.78)
**Maternal age, years**								
< 20	21/222 (9.46)	1.00 (reference)		93/304 (30.6)	1.00 (reference)		4 582/22 116 (20.7)	1.00 (reference)
20–29	172/897 (19.2)	2.12 (1.16–3.88)		420/1036 (40.5)	1.26 (0.89–1.79)		15 512/53 172 (29.2)	1.34 (1.28–1.41)
30–39	111/504 (22.0)	2.40 (1.20–4.80)		320/689 (46.4)	1.59 (1.05–2.41)		7 617/24 172 (31.5)	1.52 (1.42–1.62)
≥ 40	18/71 (25.4)	3.24 (1.30–8.06)		25/69 (36.2)	1.08 (0.55–2.15)		749/2 620 (28.6)	1.91 (1.68–2.16)
**Parity at index pregnancy^a^**								
Grand multiparous	51/218 (23.4)	1.00 (reference)		130/280 (46.4)	1.00 (reference)		1 470/5 670 (25.9)	1.00 (reference)
Multiparous	190/940 (20.2)	1.15 (0.72–1.83)		549/1260 (43.6)	1.01 (0.73–1.40)		16 695/56 559 (29.5)	1.23 (1.13–1.34)
Nulliparous	81/536 (15.1)	1.53 (0.53–4.39)		179/558 (32.1)	1.46 (0.62–3.41)		10 295/39 851 (25.8)	2.55 (2.21–2.96)
**Outcome of previous pregnancy**								
Live birth	192/971 (19.8)	1.00 (reference)		567/1292 (43.9)	1.00 (reference)		15 834/55 546 (28.5)	1.00 (reference)
Miscarriage	43/172 (25.0)	1.36 (0.78–2.34)		55/157 (35.0)	0.63 (0.39–1.00)		2 305/7 097 (32.5)	1.36 (1.25–1.48)
Stillbirth	12/42 (28.6)	2.69 (1.22–5.97)		36/68 (52.9)	1.18 (0.66–2.10)		447/940 (47.6)	1.78 (1.50–2.11)
Neonatal death	2/19 (10.5)	0.30 (0.06–1.46)		19/41 (46.3)	0.88 (0.43–1.82)		379/883 (43.0)	1.32 (1.11–1.58)
Missing data	3/9 (33.3)	3.10 (0.55–17.4)		20/31 (64.5)	1.40 (0.59–3.35)		136/564 (24.1)	1.05 (0.83–1.34)
No previous pregnancy	70/481 (14.6)	1.07 (0.40–2.88)		161/509 (31.6)	0.78 (0.34–1.77)		9 359/37 050 (25.3)	1.18 (1.04–1.34)
**No. of antenatal care visits for index pregnancy**								
0	5/36 (13.9)	1.00 (reference)		16/40 (40.0)	1.00 (reference)		255/1260 (20.2)	1.00 (reference)
1–3	138/823 (16.8)	1.27 (0.37–4.40)		409/984 (41.6)	0.96 (0.47–1.98)		8 425/35 624 (23.6)	0.97 (0.82–1.14)
4–7	170/805 (21.1)	1.63 (0.47–5.68)		416/1034 (40.2)	0.88 (0.42–1.81)		18 608/62 144 (29.9)	1.19 (1.01–1.41)
≥ 8	9/30 (30.0)	2.81 (0.61–13.0)		17/40 (42.5)	1.01 (0.38–2.73)		1 172/3 052 (38.4)	1.42 (1.18–1.72)
**Previous caesarean section^b^**	78/163 (47.9)	6.49 (4.26–9.89)		188/256 (73.4)	5.44 (3.90–7.57)		10 089/13 253 (76.1)	16.2 (15.4–17.1)
**Multiple pregnancy^b^**	37/147 (25.2)	1.29 (0.78–2.15)		75/181 (41.4)	0.97 (0.67–1.40)		2 555/7 089 (36.0)	1.24 (1.16–1.33)
**Hypertensive disorder^b^**	82/346 (23.7)	1.02 (0.71–1.46)		164/371 (44.2)	1.15 (0.87–1.50)		3 972/7 221 (55.0)	2.47 (2.33–2.63)
**Antepartum haemorrhage^b^**	58/102 (56.9)	7.48 (4.58–12.2)		212/328 (64.6)	4.86 (3.61–6.55)		827/978 (84.6)	19.0 (15.7–23.1)
**Diabetes^b^**	2/7 (28.6)	0.91 (0.12–6.99)		11/17 (64.7)	3.44 (0.97–12.2)		137/283 (48.4)	1.22 (0.91–1.63)
**Malaria^b^**	8/46 (17.4)	1.19 (0.50–2.82)		6/18 (33.3)	0.69 (0.23–2.08)		381/1 048 (36.4)	1.23 (1.06–1.43)
**Cardiac or renal disease^b^**	2/9 (22.2)	0.63 (0.09–4.27)		6/8 (75.0)	1.28 (0.20–8.20)		103/204 (50.5)	1.38 (0.96–1.97)
**Gestational diabetes^b^**	3/5 (60.0)	9.51 (0.93–96.8)		9/13 (69.2)	2.51 (0.55–11.5)		92/195 (47.2)	1.00 (0.69–1.44)
**HIV test result**								
Negative	268/1395 (19.2)	1.00 (reference)		701/1710 (41.0)	1.00 (reference)		24 849/89 595 (27.7)	1.00 (reference)
Positive	3/29 (10.3)	0.36 (0.08–1.56)		20/50 (40.0)	1.03 (0.53–1.99)		746/3 076 (24.3)	0.96 (0.86–1.06)
Not known or test not done	51/270 (18.9)	0.92 (0.61–1.39)		137/338 (40.5)	1.22 (0.92–1.62)		2 865/9 409 (30.5)	0.85 (0.80–0.90)
**Referred from another facility^b^**	182/771 (23.6)	1.98 (1.40–2.80)		492/1157 (42.5)	1.44 (1.13–1.83)		7 818/18 507 (42.2)	2.17 (2.08–2.27)
**Fetal presentation**								
Cephalic	240/1473 (16.3)	1.00 (reference)		685/1758 (39.0)	1.00 (reference)		25 744/97 007 (26.5)	1.00 (reference)
Breech	65/198 (32.8)	2.81 (1.88–4.20)		140/302 (46.4)	1.56 (1.17–2.07)		2 425/4 708 (51.5)	2.61 (2.42–2.82)
Transverse	17/23 (73.9)	28.3 (9.70–82.4)		33/38 (86.8)	16.5 (6.02–45.3)		291/365 (79.7)	15.2 (11.5–20.0)
**Macrosomia (birth weight ≥ 4000 g)^b^**	15/45 (33.3)	2.55 (1.22–5.31)		45/68 (66.2)	2.92 (1.65–5.16)		936/2 741 (34.2)	1.71 (1.55–1.88)
**Gestational age**								
Term (37–41 weeks)	181/852 (21.2)	1.00 (reference)		566/1323 (42.8)	1.00 (reference)		22 977/84 411 (27.2)	1.00 (reference)
Very preterm (< 32 weeks)	34/265 (12.8)	0.50 (0.31–0.80)		45/173 (26.0)	0.30 (0.20–0.46)		352/1 342 (26.2)	0.46 (0.39–0.54)
Moderate to late preterm (32–36 weeks)	98/535 (18.3)	0.66 (0.47–0.93)		202/529 (38.2)	0.61 (0.48–0.78)		3 935/12 655 (31.1)	0.81 (0.77–0.86)
Post-term (≥ 42 weeks)	9/42 (21.4)	1.15 (0.50–2.64)		45/73 (61.6)	2.52 (1.47–4.33)		1 196/3 672 (32.6)	1.37 (1.26–1.49)
**Country**								
Malawi	31/343 (9.04)	1.00 (reference)		106/339 (31.3)	1.00 (reference)		6 382/36 648 (17.4)	1.00 (reference)
Benin	155/688 (22.5)	1.65 (0.97–2.80)		374/936 (40.0)	1.00 (0.71–1.42)		9 729/21 447 (45.4)	1.34 (1.27–1.41)
Uganda	98/500 (19.6)	1.92 (1.15–3.21)		314/697 (45.1)	1.46 (1.05–2.03)		7 328/26 672 (27.5)	1.35 (1.29–1.41)
United Republic of Tanzania	38/163 (23.3)	4.03 (2.14–7.59)		64/126 (50.8)	2.54 (1.54–4.18)		5 021/17 313 (29.0)	1.66 (1.58–1.75)

aOR: adjusted odds ratio; CI: confidence interval; HIV: human immunodeficiency virus.

^a^ Multiparous women had had 1–4 previous deliveries and grand multiparous women had had ≥ 5.

^b^ aOR are for the comparison with births in the absence of this characteristic.

## Discussion

Our cross-sectional study of 16 hospitals in Benin, Malawi, Uganda and the United Republic of Tanzania found that around four in 10 intrapartum stillbirths were of babies born by caesarean section. Moreover, for over one quarter of intrapartum stillbirths of babies delivered by caesarean section, previous caesarean section was reported as an indication. Among antepartum stillbirths, fetal death was reported as an indication for over one third of caesarean sections. The absence of information on fetal heartbeat at admission more than doubled the estimated odds of a caesarean section among both antepartum and intrapartum stillbirths, and increased the odds by over nine times among live births. Correspondingly, the absolute risk of a caesarean section delivery was 45.5% (86/189) among antepartum stillbirths for which no information on fetal heartbeat was available at admission, 61.6% (207/336) among similar intrapartum stillbirths and 80.3% (7019/8740) of among similar live births. 

Our finding that the rate of caesarean section births was high among intrapartum stillbirths is in line with other studies from sub-Saharan Africa. One study in Zanzibar reported that the caesarean section birth rate was almost 30% for stillbirths and just over 10% for live births.^14^ In addition, a case–control study in Mozambique on the prevention of stillbirths in facilities found that caesarean sections were significantly more common among stillbirths than live births.^13^ Similarly, a population-based study in Ghana reported that the caesarean section rate among women who had a stillborn baby or whose baby died within the first day of life was double the rate among women whose baby survived the first 24 hours.^12^

We believe there are two potential explanations for our finding that the caesarean section rate was high among intrapartum stillbirths. First, the high rate could reflect a lack of timely access to the procedure due to delays between the decision to perform a caesarean section and it being done.^12^ Second, health workers might opt for a caesarean section in an attempt to save the baby’s life when the fetal heartbeat is unclear. Moreover, our analysis suggests that the high caesarean section rate among all stillbirths could be driven by the high rate among intrapartum stillbirths, which indicates that improvements in intrapartum care may be needed.

In our study, the most frequently reported indication for caesarean section overall was a history of a previous caesarean section. This finding aligns with the literature on caesarean section rates among stillbirths.^36^^–^^39^ A previous caesarean section is not an absolute indication for a repeat caesarean section, and a trial of labour has been recommended when there is no other indication.^40^ However, this approach requires close monitoring and hospitals in sub-Saharan Africa might be understaffed and under-resourced for a safe trial of vaginal delivery after a previous caesarean section.^41^ Our finding is important given that a caesarean section birth can increase both the risk of maternal illness or death, and the risk that caesarean section will be repeated in subsequent pregnancies.^42^^,^^43^ Moreover, the risk of complications increases with each procedure.

Among antepartum stillbirths in our data set, the most frequently reported indication for caesarean section was intrauterine fetal death. However, our data collection tool allowed for the reporting of more than one indication, and we found that fetal death was not the only reason reported for performing a caesarean section in over 60% of these cases. Nevertheless, the high proportion of antepartum stillbirths for which no fetal heartbeat was detected at admission indicates that facility staff either considered fetal death a valid indication for caesarean section, or made a retrospective report of fetal death to avoid blame or administrative consequences.^44^ This second reason could compromise accurate documentation and limit understanding of the underlying causes of death, thereby impairing future efforts on quality improvement, which highlights why it is crucial to promote a supportive working environment. Further investigation is needed to better understand: (i) the influence of maternal indications on decision-making by health workers; and (ii) the main reasons for deciding to perform a caesarean section, particularly if those reasons differ from the ones reported by health workers or were not captured in our data set (e.g. fear of blame).

We found that the absence of information on fetal heartbeat at admission more than doubled the odds of a caesarean section delivery among both antepartum and intrapartum stillbirths and increased the odds over nine times among live births. In line with our findings, a cohort study in the United Republic of Tanzania reported that a quarter of stillbirths of babies with no fetal heartbeat at admission were delivered by caesarean section.^45^ We suspect that uncertainty about fetal heartbeat monitoring could have contributed to the overuse of caesarean section in our study population; however, further investigations are needed. In support of our conjecture, qualitative research in northern Uganda revealed that uncertainty about fetal health increased stress among health workers.^18^ Working under stressful conditions could affect the decision-making process and lead to a caesarean section being performed despite the lack of an indication in an attempt to save the baby’s life.

Adhering to current fetal heartbeat monitoring guidelines has been reported to be challenging in sub-Saharan Africa; maternity care providers describe the lack of staff and equipment as the main obstacle to conducting adequate intermittent auscultation.^18^ In view of our finding that no information on fetal heartbeat was available for a quarter of births by caesarean section, we recommend that fetal heartbeat monitoring guidelines should be reviewed and should include recommendations on effective and feasible monitoring practices for sub-Saharan Africa.

The main strength of our study was the use of high-quality, electronic, registry data from 16 hospitals supported by rigorous data management procedures that ensured few data were missing and that the variables recorded were highly consistent.^20^

We acknowledge several limitations to the interpretation of our results. First, the possibility that the fetal heartbeat was monitored but not documented in emergencies could have biased our findings. Although this is possible, given the high case load and limited monitoring tools, it is likely that monitoring was not routinely conducted during emergencies, as has been observed in studies from similar settings.^18^ Second, the use of fetal appearance to establish the time of death is inaccurate.^46^^,^^47^ Findings from our sensitivity analysis revealed that misclassification of the time of death weakened the strength of our logistic regression models’ findings. However, we used fetal appearance because of the difficulty of establishing the start of labour in our study areas, given delays in accessing hospital care and the frequent absence of fetal heartbeat monitoring at admission. Third, although our data collection tool included internationally agreed indications for caesarean section, it also allowed more than one indication to be recorded,^48^ which limited our ability to determine the main reason for each caesarean section. Fourth, we could not adjust for potentially important confounders that were not captured in the data registry, such as socioeconomic characteristics and maternal health indicators. Thus, we must assume residual confounding. Finally, our analysis included data from four African countries, which contributed to the generalizability of our findings. However, comparisons should consider the hospital-based nature of our data.

In conclusion, we observed that birth by caesarean section in four African countries was more likely when fetal heartbeat at admission was not reported. Although we cannot exclude the possibility that the heartbeat was assessed but not recorded in some cases, our findings suggest that health workers might have performed caesarean sections to avoid risk to the fetus rather than because there was a clear medical indication. Deciding about the mode of birth is, however, a complex process, particularly when a stillbirth is possible. We believe that the development of locally tailored guidelines on the mode of birth for stillbirths that include recommendations on feasible fetal heartbeat monitoring require better understanding of fetal heartbeat monitoring and reporting practices, and of the decision-making process for caesarean section involving stillbirths.
